# Adeno-Associated Virus-Mediated Gain-of-Function mPCSK9 Expression in the Mouse Induces Hypercholesterolemia, Monocytosis, Neutrophilia, and a Hypercoagulative State

**DOI:** 10.3389/fcvm.2021.718741

**Published:** 2021-09-22

**Authors:** Georgios Louloudis, Samuele Ambrosini, Francesco Paneni, Giovanni G. Camici, Dietmar Benke, Jan Klohs

**Affiliations:** ^1^Institute for Biomedical Engineering, University of Zurich and ETH Zurich, Zurich, Switzerland; ^2^Zurich Neuroscience Center (ZNZ), Zurich, Switzerland; ^3^Center for Molecular Cardiology, University of Zurich, Zurich, Switzerland; ^4^University Heart Center, Cardiology, University Hospital Zurich, Zurich, Switzerland; ^5^Department of Research and Education, University Hospital Zurich, Zurich, Switzerland; ^6^Institute of Pharmacology and Toxicology, University of Zurich, Zurich, Switzerland

**Keywords:** PCSK9, hypercholesterolemia, mouse, neutrophils, monocytes, coagulation

## Abstract

Hypercholesterolemia has previously been induced in the mouse by a single intravenous injection of adeno-associated virus (AAV)-based vector harboring gain-of-function pro-protein convertase subtilisin/kexin type 9. Despite the recent emergence of the PCSK9-AAV model, the profile of hematological and coagulation parameters associated with it has yet to be characterized. We injected 1.0 × 10^11^ viral particles of mPCSK9-AAV or control AAV into juvenile male C57BL/6N mice and fed them with either a Western-type high-fat diet (HFD) or standard diet over the course of 3 weeks. mPCSK9-AAV mice on HFD exhibited greater plasma PCSK9 concentration and lower low-density lipoprotein levels, concomitant with increased total cholesterol and non-high-density lipoprotein (non-HDL)-cholesterol concentrations, and lower HDL-cholesterol concentrations than control mice. Furthermore, mPCSK9-AAV-injected mice on HFD exhibited no signs of atherosclerosis at 3 weeks after the AAV injection. Hypercholesterolemia was associated with a thromboinflammatory phenotype, as neutrophil levels, monocyte levels, and neutrophil-to-lymphocyte ratios were higher and activated partial thromboplastin times (aPTTs) was lower in HFD-fed mPCSK9-AAV mice. Therefore, the mPCSK9-AAV is a suitable model of hypercholesterolemia to examine the role of thromboinflammatory processes in the pathogenesis of cardiovascular and cerebrovascular diseases.

## Introduction

Hypercholesterolemia is a major risk factor for cardiovascular and cerebrovascular diseases and is associated with high mortality and morbidity (1). High lipoprotein blood levels promote atherosclerosis, which involves plaque formation in arteries by inflammation, lipid accumulation, cell death, and fibrosis (2). Clinical complications of atherosclerosis can arise from plaques causing stenosis or follow the rupture of a plaque, which exposes the pro-thrombotic material in the plaque to the blood and causes sudden thrombotic occlusion of the artery. In the heart, atherosclerosis can lead to myocardial infarction and heart failure (3); whereas in the brain, it can cause transient ischemic attacks and ischemic stroke (4). It has been suggested that hypercholesterolemia can induce and perpetuate a thromboinflammatory state (5, 6) and can thus, even in the absence of atherosclerosis, contribute significantly to the pathogenesis of cardiovascular (3) and cerebrovascular diseases (4). A release and activation of blood cells, e.g., monocytes, macrophages, platelets, endothelial cells, and T lymphocytes, together with an altered production of adhesion molecules and integrins leads to an imbalance between pro-coagulant and anti-coagulant molecules, an increased production of cytokines (e.g., interleukin-1β, interleukin-4, interleukin-6, and interleukin-10, tumor necrosis factor, and interferons), and an interaction of inflammatory cells with the vessel wall, thus promoting vascular inflammation and thrombosis (7, 8). Increased levels of inflammatory and coagulation factors in the circulation have been shown to be associated with an increased risk of myocardial infarction and ischemic stroke (9–11). Moreover, hypercholesterolemia may affect outcome in these patients by interfering with thromboinflammatory pathways (12–15), though reports have yielded controversial results (16–21). Thus, a better understanding of the role of hypercholesterolemia-mediated thromboinflammation is warranted.

Several mouse models of hypercholesterolemia have been developed based on gene mutations of the low-density lipoprotein (LDL) receptor (LDLR, Ldlr^−/−^) (22) or apolipoprotein E (ApoE^−/−^) (23). Both LDLR and ApoE are involved in hepatic uptake of lipoproteins. LDLR mediates the endocytosis of cholesterol-rich LDL, while ApoE serves as its ligand, thus maintaining plasma levels of LDL (24). ApoE^−/−^ and Ldlr^−/−^ mice have increased plasma levels of total cholesterol and LDL (25), which is further increased by feeding mice with a high-fat diet (HFD), or high-cholesterol or Western-type diet (23). Both transgenic models display thromboinflammatory states as indicated by increased levels of inflammatory cell and coagulation factors (26–28). The models also develop atherosclerotic plaques in a time-dependent fashion (23, 29).

A novel mouse model of hypercholesterolemia was developed based on the mechanisms of action of pro-protein convertase subtilisin/kexin type 9 (PCSK9) (30, 31). PCSK9 is a serine protease that is produced primarily by hepatocytes, binds hepatic LDLR, and induces its intracellular degradation, thus reducing the clearance of serum LDL (32, 33). Hypercholesterolemia is induced by a single intravenous injection of recombinant adeno-associated virus (AAV) vectors bearing either gain-of-function human PCSK9-D374Y (30) or murine PCSK9-D377Y PCSK9 (31). Atherosclerotic lesions can be observed after 12 weeks in PCSK9-AAV mice on a Western-type diet, which are exacerbated in a gene dosage-dependent manner (31). At present, there is no concrete information on the inflammatory and coagulation status of the PCSK9-AAV mouse model of hypercholesterolemia. In the present paper, we characterize the thromboinflammatory phenotype of male C57BL/6N mice injected with murine PCSK9-D377Y by hematological and coagulation profiling.

## Materials and Methods

### Animals and Treatment

All procedures were carried out according to the national guidelines of the Swiss Federal Act on Animal Protection and were approved by the Cantonal Veterinary Office Zurich (permit number: ZH080/18). We confirm compliance with the NC3Rs ARRIVE guidelines on reporting of *in vivo* experiments.

In the present study, only male C57BL/6N mice (Janvier Laboratories, Le Genest-Saint-Isle, France) were used according to our animal permit, so we did not investigate the effects of gender on hematological and coagulation parameters. Mice of 8–10 weeks of age were randomly allocated to receive either ssAAV-8/2-ApoE.HCR.hAAT-mPcsk9(D377Y)-bGHp(A) (mPCSK9-AAV) or ssAAV-8/2-ApoE.HCR.hAAT-bGHp(A) (control AAV). Cages of injected animals were randomly placed on either a Western-type HFD containing 21% crude fat and 0.15% cholesterol (KLIBA NAFAG, 2480.PH.A05) or a standard diet containing 4.5% crude fat and 300 mg/kg of cholesterol (KLIBA NAFAG, 3437.PX.L15) for 21 ± 5 days. Mice were weighed every 2–3 days. Animals were housed in a temperature-controlled facility in individually ventilated cages under a 12-h light/dark cycle. Each cage housed up to five mice. Paper tissue and red Tecniplast Mouse House® (Tecniplast, Milan, Italy) shelters were placed in cages as environmental enrichments. Food and water were provided *ad libitum*.

### Single Tail-Vein Injections of Adenovirus-Associated Vectors

A single-stranded (ss) plasmid was used to encode a gain-of-function mutant of mouse PCSK9 (pAAV/D377Y-mPCSK9). pAAV/D377Y-mPCSK9 was a gift from Jacob Bentzon (Addgene, 58376). A plasmid with no open reading frame [pssAAV-2-ApoE.HCR.hAAT-bGHp(A)] was constructed from the PCSK9-encoding plasmid by the Viral Vector Facility (VVF, University of Zurich). For both vectors, expression was driven by the ApoE promoter. Viral vectors in serotype 8 capsids [ssAAV-8/2-ApoE.HCR.hAAT-mPcsk9(D377Y)-bGHp(A) and ssAAV-8/2-ApoE.HCR.hAAT-bGHp(A)] were produced by the VVF.

Mice were anesthetized with 3% isoflurane (Abbot, Cham, Switzerland) and were maintained anesthetized with 1.5% isoflurane in a mixture of O_2_ and air (200:800 ml/min) using a face mask. Lacrinorm® ophthalmic gel (Bausch and Lomb, 7570255) was applied to the eyes of all animals to prevent drying out of corneas, and the animals were kept warm with the aid of an electric heating pad. Their tails were warmed (37°C) for vasodilatation. AAVs were dissolved in lactate Ringer (Bischel, 1000329); and 1.0 × 10^11^ viral genomes of either ssAAV-8/2-ApoE.HCR.hAAT-mPcsk9(D377Y)-bGHp(A) or ssAAV-8/2-ApoE.HCR.hAAT-bGHp(A) were administered into the lateral tail vein, with the aid of 100 Sterican® 30G × 1/2″ needles (B. Braun, 4656300) and polyethylene tubing (30 m) 100′ (0.61 OD × 0.28ID) PE-10/100 (Warner Instruments LLC, Hamden, CT, USA; 64-0751). Mice were allowed to recover in a heated recovery cage (37°C).

### Cholesterol Level Measurements

Mice were anesthetized with an i.p. bolus injection of a mixture of ketamine, xylazine, and acepromazine (75/10/2 mg/kg body weight). Blood was collected from the vena cava with 100 Sterican® 23 G needles (B. Braun, 4657640) in collection tubes in the absence of anticoagulant. Samples were allowed to stand for 30 min at room temperature, and they were centrifuged at 2,000 g for 10 min, at 4°C, for serum extraction. Total cholesterol and high-density lipoprotein (HDL) cholesterol were determined by the application of enzymatic photometric assays from Roche Diagnostics to the c502 chemistry module of the COBAS8000 autoanalyzer from Roche Diagnostics (Rotkreuz, Switzerland). Non-HDL-cholesterol was calculated as the difference between the two measures.

### Hematological Analysis

Mice were anesthetized with an i.p. bolus injection of a mixture of ketamine, xylazine, and acepromazine (75/10/2 mg/kg body weight). Blood was collected from the right cardiac ventricle with 100 Sterican® 21 G needles (B. Braun, 4657527) and sampled into Microvette® 500 K3E blood collection tubes (Sarstedt, Nümbrecht Germany; 20.1341). Hematological analysis was carried out using a Sysmex XN V analyzer.

### Coagulation Parameters

Mice were anesthetized with an overdose of sodium pentobarbital (100 mg/kg). Blood was collected from the right cardiac ventricle with 100 Sterican® 23 G needles (B. Braun, 4657640) and sampled into 3.2% (1:10) sodium citrate microtubes 0.5 ml of 9NC (Sarstedt, 41.1506.002). Collection tubes were centrifuged at 1,800 g for 10 min, at 4°C, for citrated plasma extraction. Fibrinogen concentration and aPTT were determined by the use of the Stago STart Max analyzer.

### Oil-Red O Staining

Oil-Red O staining was carried out as previously described (34). Hearts were perfused with 5 ml of phosphate-buffered solution (PBS), and aortae were dissected from the sinus to the abdominal bifurcation. Aortae were then fixed with 4% paraformaldehyde in PBS overnight and stained with Oil-Red O for 1 h. The staining solution was washed away 3× with cold PBS, and pictures were taken.

### Enzyme-Linked Immunosorbent Assay

Mice were terminally anesthetized by i.p. bolus injection of a mixture of ketamine, xylazine, and acepromazine (75/10/2 mg/kg body weight). Blood was collected from the vena cava with 100 Sterican® 23 G needles (B. Braun, 4657640) and sampled into Microvette® 500 K3E blood collection tubes (Sarstedt, 20.1341). Collection tubes were centrifuged at 2,000 g for 15 min, at 4°C. Plasma was aliquoted, preserved by snap-freezing in dry ice, and stored at −80°C. Mouse EDTA-plasma samples were subjected to PCSK9 sandwich ELISA according to the manufacturer's protocol (abcam, Cambridge, UK; ab215538). Optical densities from microplate wells were acquired with Synergy HTX Multi-Mode Microplate Reader (BioTek Instruments, Inc., Winooski, VT, USA) and Gen 5™ data analysis software (BioTek). All ELISA results were processed with ElisaAnalysis.com (Leading Technology Group, Melbourne, VIC, Australia).

### Liver Homogenization and Protein Extraction Method

Livers were extracted from mice, following transcardial whole-body perfusion with ice-cold PBS, were preserved by snap-freezing in liquid nitrogen, and were stored at −80°C. For homogenization, 500 μl of radioimmunoprecipitation assay (RIPA) buffer (Thermo Fisher Scientific, Waltham, MA, USA; 89901) with 100× Halt™ Protease Inhibitor Cocktail (1:100, Thermo Fisher Scientific, 78429) was used per 10 mg of snap-frozen liver tissue. Liver tissue was allowed to sit in RIPA buffer for 10 min at 4°C and was then disrupted by mechanical homogenization for 3 min, at 1,500 rpm and 4°C (POTTER S, B. Braun, 8533032). Samples were centrifuged for 15 min, at 14,000 rpm and 4°C (Beckman Coulter, GS-15R). Supernatants were aliquoted and frozen at −80°C.

### Western Blotting

Protein determination was done using the Pierce™ BCA Protein Assay Kit (Thermo Fisher Scientific, 23225), according to the manufacturer's protocol. Liver lysates were mixed with 2× Laemmli buffer (1:2, Bio-Rad Laboratories, Hercules, CA, USA; 1610737) and 2-mercaptoethanol (50 μl per 950 μl of Laemmli buffer). They were primed for 1 h, at 37°C, and 450-rpm shaking. Whole cell protein samples (15 μg) were loaded on 5% sodium dodecyl–polyacrylamide gels, along with 5.5 μl of pre-stained protein ladder (Chameleon® Duo, LI-COR Biosciences, Lincoln, NE, USA; 928-60000), and were run in a Mini Protean Tetra System (Bio-Rad, 1658004) with Tris-glycine running buffer [25 mM of Tris, 192 mM of glycine, 0.1% (w/v) SDS]. Samples were then transferred to 0.45 μm of nitrocellulose acetate membranes (Bio-Rad, 1620115) at 15 V, for 95 min, with Tris-glycine transfer buffer [48 mM of Tris, 39 mM of glycine, 0.13 mM of SDS, 20% (v/v) methanol] using a Trans-blot SD, semi-dry transfer cell (Bio-Rad, 1703940). Membranes were stained for 5 min with Revert™ 700 Total Protein Stain and were then washed with Revert™ Wash solution [6.7% (v/v) glacial acetic acid, 30% (v/v) methanol] three times (30 s per wash) (LI-COR, 926-11010). Membranes were imaged with an Odyssey® imaging system (LI-COR Biosciences, CLx) in the 700-nm channel. Membranes were blocked in 5% non-fat dry milk in PBS for 1 h. They were probed overnight with a goat anti-mouse LDL receptor antibody (1:2,000, R&D Systems, AF2255) in TBST-Tween-20 (TBS-T, 0.05% Tween-20) solution containing 5% milk, at 4°C. Membranes were washed five times in TBS-T (10 min per wash). They were probed with donkey anti-goat IRDye® 800 CW conjugated secondary antibody (1:8,000, LI-COR Biosciences, 926-32214) in TBS-T containing 5% milk for 1 h, in the dark and at room temperature. They were washed four times with TBST-T and one time with TBS in the dark (10 min per wash). Membranes were imaged again with the Odyssey® imaging system (LI-COR Biosciences, CLx) in the 800-nm channel. Fluorescent signals were quantified using Image Studio™ Lite Ver 5.2 (LI-COR Biosciences). Preliminary experiments using increasing protein and antibody concentrations were performed to ensure measurement of signals in the linear range.

### Statistical Analysis

Data are presented as mean ± standard deviation (SD). Comparisons were made by two-way ANOVA followed by Tukey's test. Repeated-measures ANOVA followed by the Holm–Sidak test was used for comparisons of body weights across the study period. Unpaired *t*-test was used for comparisons of relative levels of hepatic Ldlr.

## Results

### Elevated PCSK9 Plasma Levels and Hypercholesterolemia Induced by mPCSK9-AAV Expression and Intake of Western-Type High-Fat Diet

**Figure 1:**
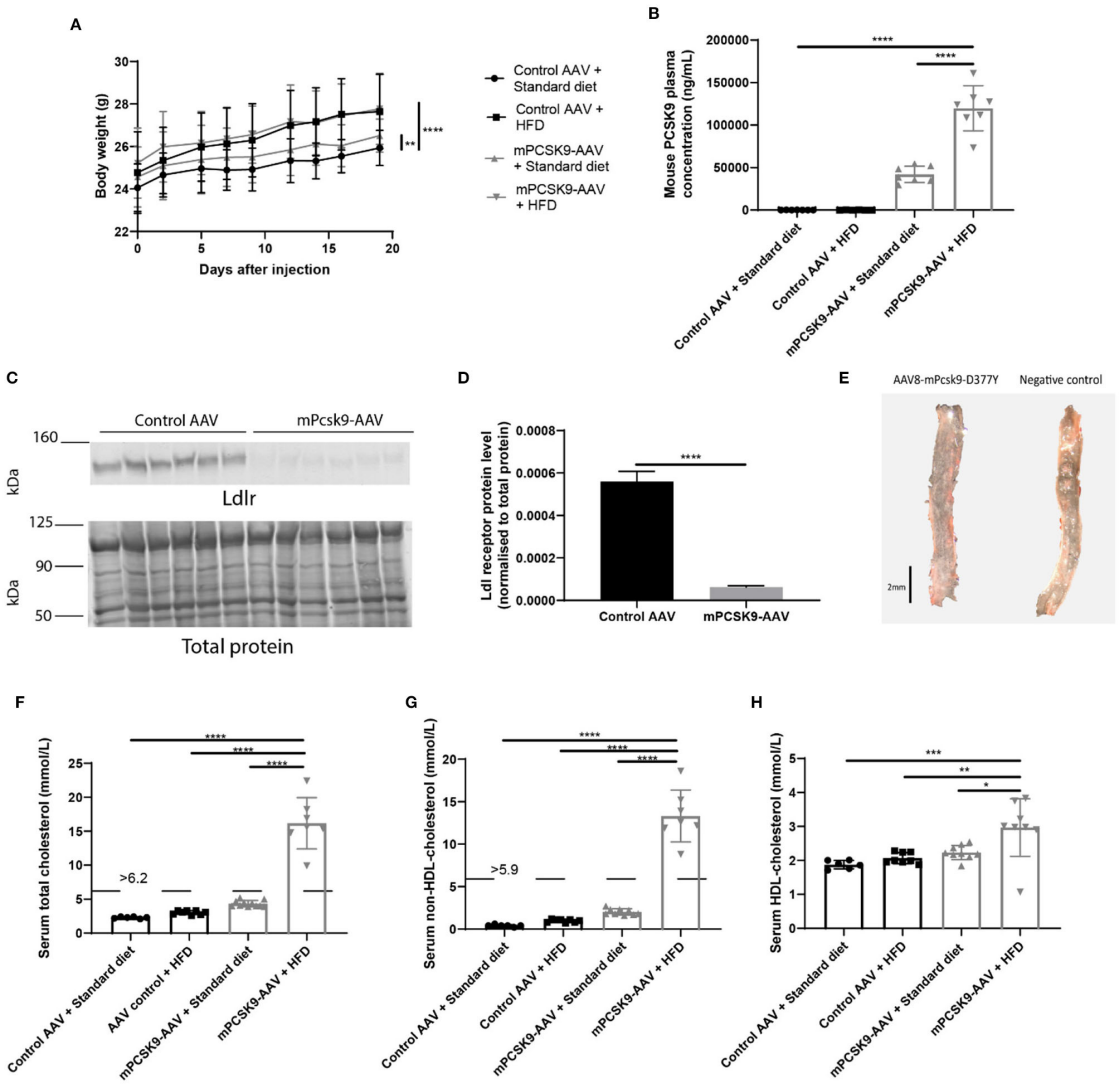
Verification of the mPCSK9-AAV mouse model of hypercholesterolemia. **(A)** Body weights of the four experimental groups: Control AAV + Standard diet (black circles, *n* = 12), Control AAV + HFD (black squares, *n* = 10), mPCSK9-AAV + Standard diet (gray upward triangles, *n* = 11), and mPCSK9-AAV + HFD (gray downward triangles, *n* = 10). Mean ± SD; repeated-measures ANOVA with Holm–Sidak *post-hoc* test; **p ≤ 0.01, ****p ≤ 0.0001. **(B)** Mouse PCSK9 plasma concentration in Control AAV + Standard diet (circles, *n* = 7), Control AAV + HFD (squares, *n* = 6), mPCSK9-AAV + Standard diet (upward triangles, *n* = 7), and mPCSK9-AAV + HFD (downward triangles, *n* = 7) groups. Data are the mean of three independent replicates. Mean ± SD; two-way ANOVA with Tukey's *post-hoc* test; ****p ≤ 0.0001. **(C,D)** Hepatic Ldl receptor (Ldlr) levels in control-injected (*n* = 6) and mPCSK9-AAV-injected mice (*n* = 6) on standard diet normalized to total protein. Data are the mean of three independent replicates. Two gels were loaded per replicate. Mean ± SD; unpaired *t*-test; ****p ≤ 0.0001. **(E)** Representative images of Oil-red O-stained thoracic aortae of mPCSK9-AAV and control AAV mice on HFD. Mice did not develop atherosclerotic lesions in the selected time frame. Scale bar, 2 mm. **(F–H)** Serum total cholesterol **(F)**, non-HDL cholesterol **(G)**, and HDL cholesterol **(H)** in the different groups: Control AAV + Standard diet (*n* = 6), Control AAV + HFD (n ≥ 8), mPCSK9-AAV + Standard diet (*n* ≥ 9), and mPCSK9-AAV + HFD (*n* ≥ 7). Mean ± SD; two-way ANOVA with Tukey's *post-hoc* test; *p ≤ 0.05, **p ≤ 0.01, ***p ≤ 0.001, ****p ≤ 0.0001. AAV, adeno-associated virus; HFD, high-fat diet; HDL, high-density lipoprotein.

To verify whether hypercholesterolemia is driven by mPCSK9-AAV gene expression, we injected mice with 1.0 × 10^11^ viral genomes of either mPCSK9-AAV or control AAV; and we then fed them with either a Western-type HFD or standard diet for a total of 21 ± 2 days. Body weight was monitored regularly during that period (Figure 1A). Mice fed with the HFD irrespective of the injected vector significantly gained more weight than mice that received the standard diet. Notably, injection of mPCSK9-AAV in mice on the standard diet led also to significant increased weight gains in comparison with control AAV-injected mice on the standard diet. To assess if we can specifically induce mPCSK9 overexpression by AAV injection, we assessed mPCSK9 plasma levels on day 21 after injection (Figure 1B). The mPCSK9-AAV-injected mice on the HFD displayed 847-fold higher plasma mouse PCSK9 levels as compared with the control AAV-injected mice on the standard diet, and 799-fold higher plasma mouse PCSK9 levels compared with the control AAV-injected mice on the HFD. The mPCSK9-AAV-injected mice on the standard diet had a 297-fold higher plasma mouse PCSK9 levels compared with the control AAV-injected mice on the standard diet, and 280-fold increased PCSK9 plasma levels compared with the control-AAV-injected mice on the HFD. However, PCSK9 levels were significantly higher in HFD-fed mice compared with standard diet-fed mice, hinting at a potential role that the diet may have exerted in the elevation of PCSK9 levels.

PCSK9 binds hepatic LDLR directing them for degradation in lysosomes (35). Thus, we next examined hepatic Ldlr protein levels to determine how effective the adenoviral-mediated expression of mPCSK9 depletes Ldlr (Figures 1C,D). We observed a nine-fold decrease in relative Ldlr protein level in mPCSK9-AAV compared with control AAV-injected animals. To verify the absence of atherosclerotic lesions in the selected time frame of hypercholesterolemia, we dissected aortic samples from HFD mice, 21 days following i.v. injection of mPcsk9-D377Y-AAV or control AAV vector for Oil-Red O staining. Neither controls AAV nor mice on the mPcsk9-D377Y-AAV8 vector exhibited atherosclerotic lesions (Figure 1E). Furthermore, mPCSK9-AAV injected mice on the HFD had elevated total, HDL-cholesterol and non-HDL cholesterol serum levels (Figures 1F–H). mPCSK9-AAV-injected mice on the standard diet and mice injected with control AAV on the HFD had both elevated total and non-HDL serum cholesterol levels. However, only mice injected with mPSCK9-AAV on the HFD displayed hypercholesterolemia.

### Hypercholesterolemia Is Associated With Neutrophilia and Monocytosis

**Figure 2:**
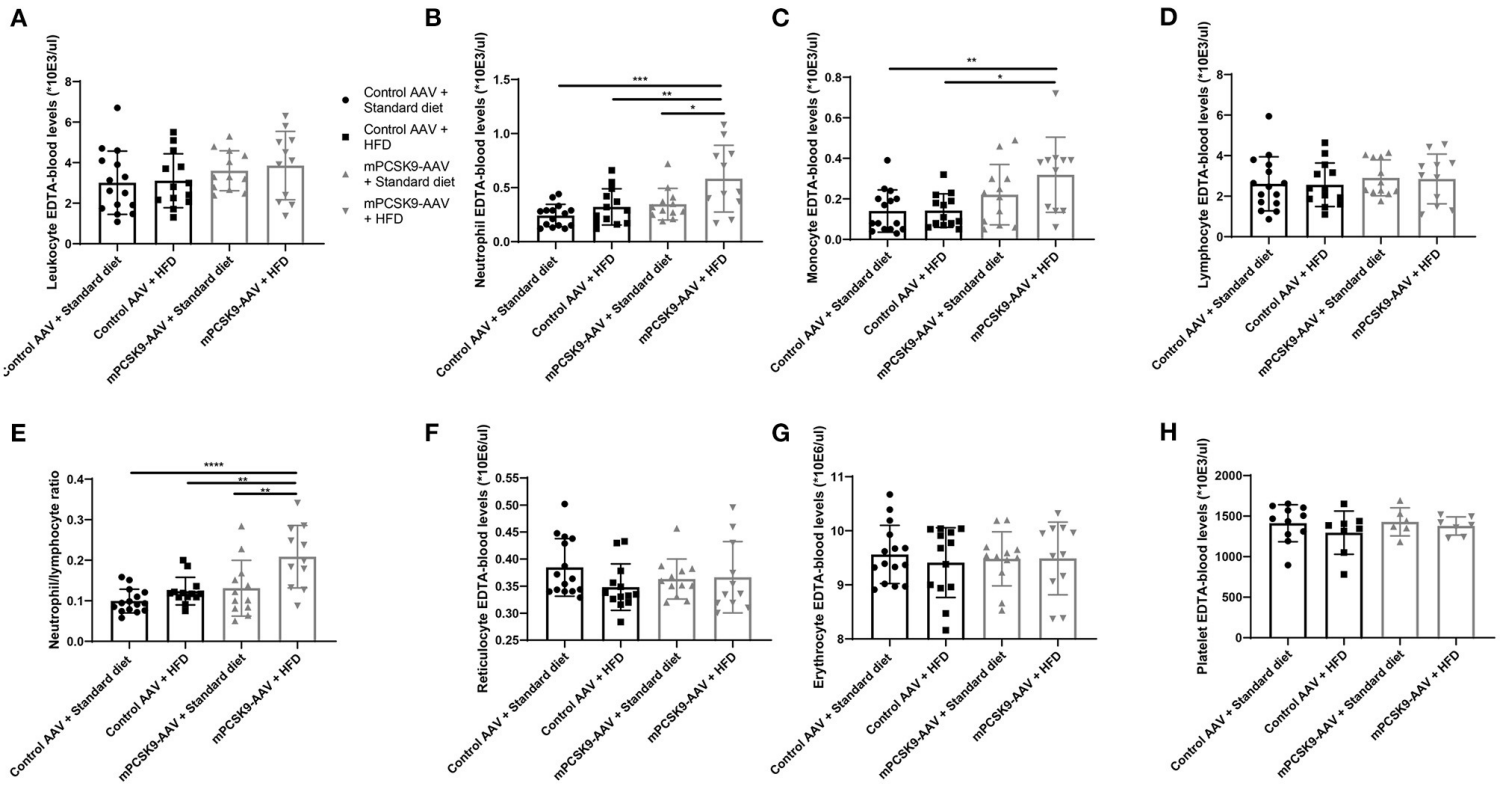
Hematological profiling of the mPCSK9-AAV mouse model of hypercholesterolemia. Leukocytes **(A)**, neutrophils **(B)**, monocytes **(C)**, lymphocytes **(D)**, neutrophil-to-lymphocyte ratio **(E)**, reticulocyte **(F)**, and erythrocyte **(G)** levels in EDTA-blood of the experimental groups: Control AAV + Standard diet (*n* = 15, circles), Control AAV + HFD (*n* = 13, squares), mPCSK9-AAV + Standard diet (*n* = 12, upward triangles), and mPCSK9-AAV + HFD (*n* = 11, downward triangles). **(H)** Platelet levels in EDTA-blood of experimental groups: Control AAV + Standard diet (*n* = 11, circles), Control AAV + HFD (*n* = 8, squares), mPCSK9-AAV + Standard diet (*n* = 6, upward triangles), and mPCSK9-AAV + HFD (*n* = 7, downward triangles). Mean ± SD; two-way ANOVA with Tukey's *post-hoc* test; *p ≤ 0.05, **p ≤ 0.01, ***p ≤ 0.0005, ****p ≤ 0.0001. AAV, adeno-associated virus; HFD, high-fat diet.

To determine the effects of hypercholesterolemia on inflammatory parameters, we characterized the hematological profile of mPCSK9-AAV or control AAV mice fed with either HFD or the standard diet 21 days post-injection (Figure 2). While there were no overall differences in leukocyte levels (Figure 2A), mPCSK9-AAV mice on HFD exhibited significantly greater neutrophil levels, monocyte levels, and neutrophil-to-lymphocyte ratios (Figures 2B,C,E). Specifically, HFD-fed mPCSK9-AAV mice exhibited neutrophil levels 1.7-fold greater than did mPCSK9-AAV mice on standard diet, 1.8-fold greater than did HFD-fed control mice, and 2.4-fold greater than did control mice on standard diet. mPCSK9-AAV mice on HFD demonstrated 2.2-fold greater monocyte levels than did HFD-fed control mice and 2.3-fold greater than did control mice on standard diet. Neutrophil-to-lymphocyte ratios were 1.6-, 1.7-, and 2.1-fold greater in HFD-fed mPCSK9-AAV mice compared with mPCSK9-AAV mice on standard diet, HFD-fed control mice, and double control mice, respectively. There were no significant differences in lymphocyte, erythrocyte, leukocyte, reticulocyte, and platelet levels across all experimental groups (Figures 2D,F–H). These data indicate that hypercholesterolemia induces neutrophilia and monocytosis.

### Hypercholesterolemia Is Associated With a Reduced Activated Partial Thromboplastin Time and Increased Plasma Fibrinogen Levels

**Figure 3:**
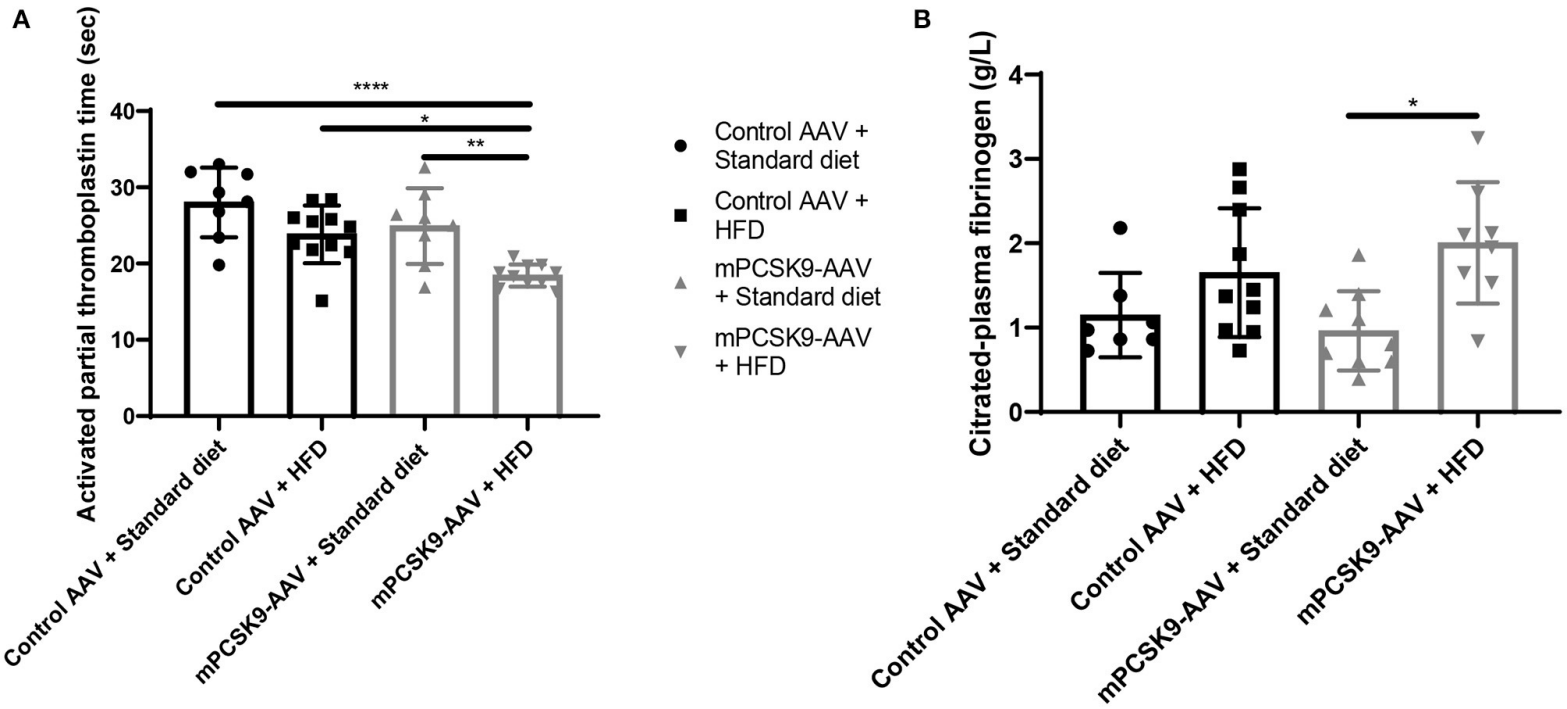
aPTT and fibrinogen levels in mice. **(A)** aPTT profiles of the four experimental groups: Control AAV + Standard diet (circles, *n* = 8), Control AAV + HFD (squares, *n* = 11), mPCSK9-AAV + Standard diet (upward triangles, *n* = 8), and mPCSK9-AAV + HFD (downward triangles, *n* = 10). **(B)** Fibrinogen levels in the four experimental groups: Control AAV + Standard diet (circles, *n* = 7), Control AAV + HFD (squares, *n* = 10), mPCSK9-AAV + Standard diet (upward triangles, *n* = 9), and mPCSK9-AAV + HFD (downward triangles, *n* = 8). Mean ± SD; two-way ANOVA with Tukey's *post-hoc* test; *p ≤ 0.05, **p ≤ 0.01, ****p ≤ 0.0001. aPTT, activated partial thromboplastin time; AAV, adeno-associated virus; HFD, high-fat diet.

To study whether PCSK9- and HFD-driven hypercholesterolemia alter coagulation parameters, we sampled blood from mice injected with either mPCSK9-AAV or control AAV and fed with either HFD or the standard diet 21 days post-injection (Figure 3). Mice either overexpressing PCSK9 or consuming HFD exhibited lower aPTT compared with control mice on standard diet, whereas mice injected with mPCSK9-AAV on HFD had the highest reduction (37.5%) in aPTT (Figure 3A). Fibrinogen levels were significantly higher in HFD-fed mPCSK9-AAV mice compared with mPCSK-AAV on standard diet (Figure 3B), while there was no significant differences in fibrinogen levels between the other groups.

## Discussion

The transgenic Ldlr^−/−^ and ApoE^−/−^ mouse lines are widely used models of hypercholesterolemia (22, 23). AAV-mediated expression of gain-of-function murine PCSK9-D377Y is a novel and increasingly used alternative mouse model (30, 31). Our data show that 3 weeks of AAV-mediated expression of murine PCSK9-D377Y in male C57BL/6N mice produces hypercholesterolemia. In addition, we show that short intervals of mPCSK9-AAV-mediated hypercholesterolemia, in the absence of atherosclerosis, induces a thromboinflammatory phenotype, previously described for the Ldlr^−/−^ and ApoE^−/−^ lines.

We systematically compared plasma murine PCSK9 and lipoprotein levels across both vector and diet groups. The mean mouse PCSK9 plasma concentration in mPCSK9-AAV mice on standard diet was approximately 42,000 ng/ml, whereas the mean murine PCSK9 concentration in mPCSK9-AAV mice on HFD was approximately 120,000 ng/ml (Figure 1B). A previous study by Bjørklund et al. reported lower plasma mouse PCSK9 concentrations in mPCSK9-AAV mice on Western-type diet (31). However, our values are in line with a study by Vozenilek et al. using the same construct (36). Differences in blood mPCSK9 expression can be attributed to gender differences in AAV-8 distribution.

Our study further showed that AAV-mediated expression of mPCSK9 depletes Ldlr in the liver (Figures 1C,D) and that if these mice are placed on a HFD, they develop hypercholesterolemia (Figures 1F–H). Mice injected with mPCSK9-AAV and fed the HFD had approximately a mean total cholesterol serum level of 16 mmol/L (Figure 1F). Concomitantly, the non-HDL-cholesterol and HDL-cholesterol concentrations were significantly higher in mPCSK9-AAV mice on HFD compared to control AAV mice on standard diet (Figures 1G,H). Although mPCSK9-AAV on standard diet and control AAV-injected mice on HFD tended to have increased total and non-HDL cholesterol levels, this did not reach significance. Thus, this demonstrates that HFD exacerbates lipoprotein levels, inducing hypercholesterolemia in mPCSK9-AAV-injected mice, in line with previous reports (30, 31). Taken together, these data confirm previous reports that injection of mPCSK9-AAV in mice results in overproduction of the gain-of-function mPCSK9, which binds the Ldlr in the liver, resulting in its degradation and a corresponding increase in serum cholesterol levels that eventually leads to hypercholesterolemia when combined with HFD (30, 31). It has been shown that a single intravenous administration of recombinant AAV supports long-term protein expression without eliciting liver damage, immunologic effects, or adverse effects in mice (37). However, despite low immunogenicity, responses of humoral immunity to AAV may still occur (38). In the present study, the control AAV consisted of the same capsid, and its expression was driven by the same promoter as that of the mPCSK9-AAV.

Increased levels of cholesterol and lipid proteins in the blood induce immunological and coagulation changes in different tissues (39, 40), which are linked to the pathogenesis of cardiovascular and cerebral diseases (7, 8). Thus, we characterized the hematological and coagulation profiles in the mPCSK9-AAV mouse model of hypercholesterolemia. Previous studies reported the effects of hypercholesterolemia on homeostasis of bone marrow-derived cells in Ldlr^−/−^ and ApoE^−/−^ mice (6, 26, 41). However, in those studies, hypercholesterolemia in transgenic mice was induced for longer periods than 3 weeks to model atherosclerosis, which might induce additional effects on inflammatory and coagulation parameters. The gain-of-function mPCSK9-D374Y model of hypercholesterolemia has so far not been characterized. We confirmed that at a time frame of 3 weeks, HFD after mPCSK9-AAV delivery does not induce atherosclerosis (Figure 1E). Thus, all observed hematological and coagulation changes can be attributed to hypercholesterolemia.

We did not observe differences in the number of leukocytes between all experimental groups but found monocytosis in mPCSK9-AAV on the HFD (Figure 2C). Hypercholesterolemia-associated monocytosis may result from continued bone marrow production of monocytes, increased survival of these cells in the periphery, and impaired conversion to resident monocytes (41). Increased monocyte levels have been associated with an increased risk of myocardial infarction (10). Moreover, increased lipoproteins levels induce an adhesive phenotype in circulating monocytes, which together with an upregulation of adhesion molecules on the endothelium leads to an accumulation of monocytes at the vessel wall, an important step in the pathogenesis of atherosclerosis (42).

In addition, we observed neutrophilia in mPCSK9-AAV-injected mice on the HFD (Figure 2B). Hypercholesterolemia in ApoE^−/−^ mice on an HFD has been shown to trigger neutrophilia by means of cholesterol-induced pro-inflammatory cytokines, enhanced granulopoiesis, and enhanced mobilization from the bone marrow (26). Lipoproteins also stimulate neutrophil adhesion (43, 44) and transendothelial migration (45) and induce the release of secondary and primary granules as indicated by discharge of lactoferrin and myeloperoxidase, respectively (45). Hypercholesterolemia modulates the pro-inflammatory effects of neutrophils, that partake in the pathogenesis of myocardial infarction and ischemic stroke (7, 8). Moreover, neutrophils are crucially involved in tissue damage following these events, which is exacerbated by hypercholesterolemia (12–14, 21).

Furthermore, we found that mPCSK9-AAV mice on HFD exhibit significantly lower aPTT values compared with each of the other three experimental groups (Figure 3A), indicative of a hypercoagulative state. Reduced aPTT together with increased concentrations of coagulation factors such as factors VIIc, VIII, VIIIc, and XII, antithrombin III, plasminogen, and serum globulin; increased plasma, serum, and blood viscosity; and a reduction in fibrinolytic activity has previously been reported in hypercholesterolemic patients (46–48). A negative correlation between circulating PCSK9 concentration and aPTT was recently noted in patients with chest pain (49). Studies in transgenic Ldlr^−/−^ and ApoE^−/−^ mice provided additional evidence for the activation of both the intrinsic and extrinsic coagulation cascades (15, 28). Furthermore, fibrinogen levels were significantly higher in HFD-fed mPCSK9-AAV mice compared with mPCSK-AAV on standard diet while not being significantly different compared with other groups (Figure 3B). Hypercholesterolemia is associated with elevated fibrinogen levels (46, 50, 51), but the effects of cholesterol-lowering therapy on fibrinogen levels remain controversial (48, 52). In a study of patients with stable coronary artery disease, fibrinogen levels tended to be higher with high circulating PCSK9 concentration (53). Similarly, PCSK9 inhibitors have had no effect on fibrinogen levels in patients with familial hypercholesterolemia (54). Taken together, the data indicate that hypercholesterolemia induced by AAV-mediated mPCSK9 expression in mice induces a hypercoagulative state. A hypercoagulative state can predispose patients for thrombosis that results in myocardial infarction or ischemic stroke (7, 8) and can be further amplified by inflammatory cells, for example, the interaction of leukocyte with the endothelium (55) or pro-thrombotic mechanisms of neutrophils (56).

The study has several limitations. Here, we provide only a very broad characterization of the thromboinflammatory phenotype of male mice injected with mPCSK9-AAC. Future studies should provide a more in-depth characterization of the molecular features of the model, such as the roles of inflammatory cell subpopulations, pro-inflammatory and anti-inflammatory cytokines, chemokines, reactive oxygen species, and inflammasomes, so as to further verify the clinical relevance of the model toward the study of human disease and monitor the therapeutic effects of anti-hypercholesterolemic drugs (48) and lifestyle adaptions (47). A major limitation of our study is that we used only male mice AAV8 targeting of liver tissue, which is more efficacious in male mice (57), while AAV-8 tissue tropism is more widely found in female mice, and this translates in higher mPCSK9 levels in male than in female mice when an mPCSK9-AAC construct is injected (36). Gender differences in the phenotype have been described for the Ldlr^−/−^ model (58). Thus, future studies should compare differences in the thromboinflammatory phenotype between mPCSK9-AAV mice of different gender.

Taken together, we have shown that gain-of-function mPCSK9-AAV model and HFD induce hypercholesterolemia and is associated with a thromboinflammatory phenotype, in the absence of atherosclerotic plaques. It provides a versatile tool for hypercholesterolemia research that robustly induced a phenotype known for ApoE^−/−^ and Ldlr^−/−^ lines (22, 23, 25), but that overcomes some of the limitations of germline genetically engineered models and can be combined with transgenic techniques and lines. The model can be useful in experimental models of cardiovascular and cerebrovascular diseases (59–61) to study the role of hypercholesterolemia in the disease pathogenesis and outcome.

## Data Availability Statement

The raw data supporting the conclusions of this article will be made available by the authors, without undue reservation.

## Ethics Statement

The animal study was reviewed and approved by Cantonal Veterinary Office Zurich.

## Author Contributions

GL and JK designed the experiments. GL, SA, and JK performed the experiments. GL, DB, and JK analyzed the data. GL and JK wrote the article. All coauthors made critical revisions to the manuscript.

## Funding

The authors disclose receipt of the following financial support for the research, authorship, and/or publication of this article: Swiss National Science Foundation (320030_179277) to JK. FP is the recipient of a Sheikh Khalifa's Foundation Assistant Professorship in Cardiovascular Regenerative Medicine at the Faculty of Medicine, University of Zurich.

## Conflict of Interest

The authors declare that the research was conducted in the absence of any commercial or financial relationships that could be construed as a potential conflict of interest.

## Publisher's Note

All claims expressed in this article are solely those of the authors and do not necessarily represent those of their affiliated organizations, or those of the publisher, the editors and the reviewers. Any product that may be evaluated in this article, or claim that may be made by its manufacturer, is not guaranteed or endorsed by the publisher.
