# Corrigendum: Adeno-Associated Virus-Mediated Gain-of-Function mPCSK9 Expression in the Mouse Induces Hypercholesterolemia, Monocytosis, Neutrophilia, and a Hypercoagulative State

**DOI:** 10.3389/fcvm.2021.820282

**Published:** 2022-01-12

**Authors:** Georgios Louloudis, Samuele Ambrosini, Francesco Paneni, Giovanni G. Camici, Dietmar Benke, Jan Klohs

**Affiliations:** ^1^Institute for Biomedical Engineering, University of Zurich and ETH Zurich, Zurich, Switzerland; ^2^Zurich Neuroscience Center (ZNZ), Zurich, Switzerland; ^3^Center for Molecular Cardiology, University of Zurich, Zurich, Switzerland; ^4^University Heart Center, Cardiology, University Hospital Zurich, Zurich, Switzerland; ^5^Department of Research and Education, University Hospital Zurich, Zurich, Switzerland; ^6^Institute of Pharmacology and Toxicology, University of Zurich, Zurich, Switzerland

**Keywords:** PCSK9, hypercholesterolemia, mouse, neutrophils, monocytes, coagulation

In the original article, there was a mistake in Figure 1H as published. The graph incorrectly depicting higher serum HDL-cholesterol levels in control AAV mice on standard diet compared to the rest of the experimental groups. The corrected Figure 1H appears below. It depicts higher serum HDL-cholesterol levels in the mPCSK9-AAV mice on HFD compared to the rest of our experimental groups.

A correction has been made to **Results, Elevated PCSK9 plasma levels and hypercholesterolemia induced by mPCSK9-AAV expression and intake of Western-type HFD, paragraph 2**:

“Furthermore, mPCSK9-AAV injected mice on the HFD had elevated total, HDL-cholesterol and non-HDL cholesterol serum levels (Figures 1F–H).”

**Figure 1:**
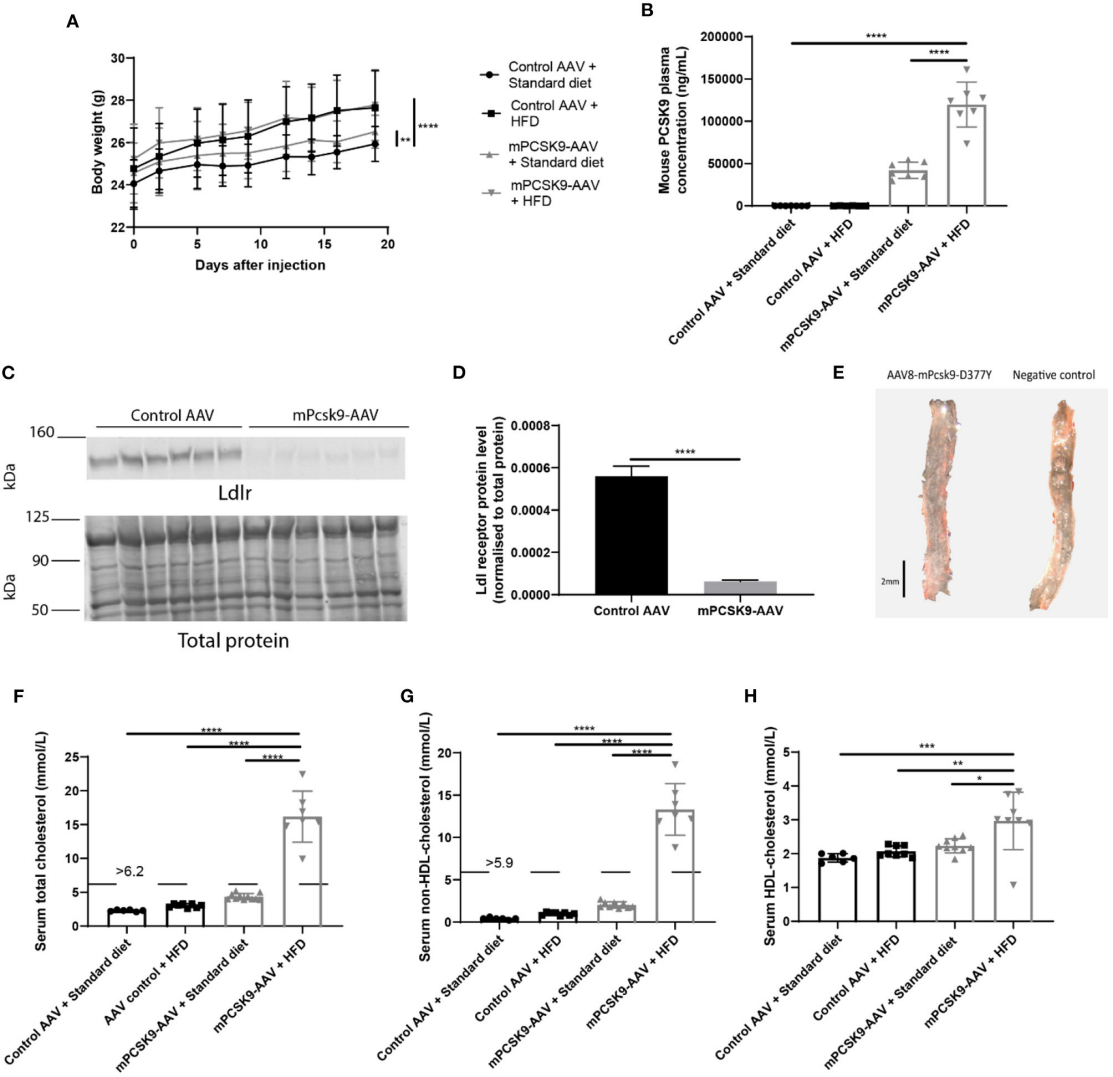
Verification of the mPCSK9-AAV mouse model of hypercholesterolemia. **(A)** Body weights of the four experimental groups: Control AAV + Standard diet (black circles, *n* = 12), Control AAV + HFD (black squares, *n* = 10), mPCSK9-AAV + Standard diet (gray upward triangles, *n* = 11), and mPCSK9-AAV + HFD (gray downward triangles, *n* = 10). Mean ± SD; repeated-measures ANOVA with Holm–Sidak *post-hoc* test;^**^p ≤ 0.01, ^****^p ≤ 0.0001. **(B)** Mouse PCSK9 plasma concentration in Control AAV + Standard diet (circles, *n* = 7), Control AAV + HFD (squares, *n* = 6), mPCSK9-AAV + Standard diet (upward triangles, *n* = 7), and mPCSK9-AAV + HFD (downward triangles, *n* = 7) groups. Data are the mean of three independent replicates. Mean ± SD; two-way ANOVA with Tukey's *post-hoc* test; ^****^p ≤ 0.0001. **(C,D)** Hepatic Ldl receptor (Ldlr) levels in control-injected (*n* = 6) and mPCSK9-AAV-injected mice (*n* = 6) on standard diet normalized to total protein. Data are the mean of three independent replicates. Two gels were loaded per replicate. Mean ± SD; unpaired *t*-test; ^****^p ≤ 0.0001. **(E)** Representative images of Oil-red O-stained thoracic aortae of mPCSK9-AAV and control AAV mice on HFD. Mice did not develop atherosclerotic lesions in the selected time frame. Scale bar, 2 mm. **(F–H)** Serum total cholesterol **(F)**, non-HDL cholesterol **(G)**, and HDL cholesterol **(H)** in the different groups: Control AAV + Standard diet (*n* = 6), Control AAV + HFD (n ≥ 8), mPCSK9-AAV + Standard diet (*n* ≥ 9), and mPCSK9-AAV + HFD (*n* ≥ 7). Mean ± SD; two-way ANOVA with Tukey's *post-hoc* test; ^*^p ≤ 0.05, ^**^p ≤ 0.01, ^***^p ≤ 0.001, ^****^p ≤ 0.0001. AAV, adeno-associated virus; HFD, high-fat diet; HDL, high-density lipoprotein.

Furthermore, a correction has been made to the **Discussion, paragraph 3:**

“Concomitantly, the non-HDL-cholesterol and HDL-cholesterol concentrations were significantly higher in mPCSK9-AAV mice on HFD compared to control AAV mice on standard diet (Figures 1G,H).”

The authors apologize for this error and state that this does not change the scientific conclusions of the article in any way. The original article has been updated.

## Publisher's Note

All claims expressed in this article are solely those of the authors and do not necessarily represent those of their affiliated organizations, or those of the publisher, the editors and the reviewers. Any product that may be evaluated in this article, or claim that may be made by its manufacturer, is not guaranteed or endorsed by the publisher.

